# First-time Mothers’ Understanding and Use of a Pregnancy and Parenting Mobile App (The Baby Buddy App): Qualitative Study Using Appreciative Inquiry

**DOI:** 10.2196/32757

**Published:** 2022-11-21

**Authors:** Elizabeth Bailey, Samantha Nightingale, Nicky Thomas, Dawn Coleby, Toity Deave, Trudy Goodenough, Samuel Ginja, Raghu Lingam, Sally Kendall, Crispin Day, Jane Coad

**Affiliations:** 1 Coventry University Coventry United Kingdom; 2 Elizabeth Bryan Multiple Births Centre Centre for Social Care, Health and Related Research Birmingham City University Birmingham United Kingdom; 3 University Hospitals Coventry and Warwickshire NHS Trust Coventry United Kingdom; 4 George Eliot NHS Trust Nuneaton United Kingdom; 5 Division of Health Sciences Warwick Medical School University of Warwick Coventry United Kingdom; 6 School of Nursing and Midwifery Faculty of Health and Life Sciences De Montfort University Leicester United Kingdom; 7 School of Health and Social Wellbeing University of the West of England Bristol United Kingdom; 8 School of Psychology Ulster University Ulster United Kingdom; 9 University of New South Wales Sydney Australia; 10 Centre for Health Services Studies University of Kent Canterbury United Kingdom; 11 Institute of Psychiatry, Psychology and Neuroscience Kings College London London United Kingdom; 12 School of Health Sciences Faculty of Medicine & Health Sciences University of Nottingham Nottingham United Kingdom

**Keywords:** pregnancy, antenatal support, antenatal education, communication, digital, pregnancy apps, mobile phone

## Abstract

**Background:**

Internationally, there is increasing emphasis on early support for pregnant women to optimize the health and development of mothers and newborns. To increase intervention reach, digital and app-based interventions have been advocated. There are growing numbers of pregnancy health care apps with great variation in style, function, and objectives, but evidence about impact on pregnancy well-being and behavior change following app interaction is lacking. This paper reports on the qualitative arm of the independent multicomponent study exploring the use and outcomes of first-time mothers using the Baby Buddy app, a pregnancy and parenting support app, available in the National Health Service App Library and developed by a UK child health and well-being charity, Best Beginnings.

**Objective:**

This study aims to understand when, why, and how first-time mothers use the Baby Buddy app and the perceived benefits and challenges.

**Methods:**

This paper reports on the qualitative arm of an independent, longitudinal, mixed methods study. An Appreciative Inquiry qualitative approach was used with semistructured interviews (17/60, 28%) conducted with new mothers, either by telephone or in a focus group setting. First-time mothers were recruited from 3 study sites from across the United Kingdom. Consistent with the Appreciative Inquiry approach, mothers were prompted to discuss what worked well and what could have been better regarding their interactions with the app during pregnancy. Thematic analysis was used, and findings are presented as themes with perceived benefits and challenges.

**Results:**

The main benefit, or what worked well, for first-time mothers when using the app was being able to access new information, which they felt was reliable and easy to find. This led to a feeling of increased confidence in the information they accessed, thus supporting family and professional communication. The main challenge was the preference for face-to-face information with a health care professional, particularly around specific issues that they wished to discuss in depth. What could have been improved included that there were some topics that some mothers would have preferred in more detail, but in other areas, they felt well-informed and thus did not feel a need to seek additional information via an app.

**Conclusions:**

Although this study included a small sample, it elicited rich data and insights into first-time mothers’ app interactions. The findings suggest that easily accessible pregnancy information, which is perceived as reliable, can support first-time mothers in communicating with health care professionals. Face-to-face contact with professionals was preferred, particularly to discuss specific and personalized needs. Further studies on maternal and professional digital support preferences after the COVID-19 global pandemic and how they facilitate antenatal education and informed decision-making are recommended, particularly because digital solutions remain as a key element in pregnancy and early parenting care.

**International Registered Report Identifier (IRRID):**

RR2-10.1017/S1463423618000294

## Introduction

### Background

The widespread accessibility and ease of communication have underpinned the development of *mobile apps*, which have led to a fundamental change in the methods that people use to access information [1]. OFCOM [2] described apps that present information in a “streamlined way that allowed them to access details they needed quickly.” In 2021, the estimated number of global smartphone users was 6.4 billion, 73.9% increase from 2016 and growth rate that will mean that, by 2023, more than half the people in the world will own a smartphone [3]. In 2020, 429 pregnancy apps were identified in the Apple App Store and 1006 in the Android equivalent platform, Google Play Store [4]

Previous literature on the design, content, and application of pregnancy apps highlights that although there are high number of apps targeting pregnancy, quality and usability are highly varied, as are the approaches and outcome measures used to evaluate their impact [5]. When the complexity of pregnancy is considered in the context of app design and interaction, it has been observed that most apps use a shallow functionality with static information framed toward a medical view of pregnancy care [6]. As pregnancy presents physical, informational, emotional, and social needs, designers need to consider how to provide support beyond static information or direction to medical professionals. The five common *types* of pregnancy app content have been identified [6] and include the following: (1) reference encyclopedias, (2) guides tied to pregnancy timelines or daily information, (3) tracker logs or countdowns, (4) heads-up dashboards, and (5) text or photograph journal functions. Following analysis of identified pregnancy apps in which self-care, psychosocial peer support, and partner or spouse support were largely absent, Peyton and Wisniewski [6] suggested that app designers consider the social experience of pregnancy beyond the medical and corporeal context.

In general, women seek to use perinatal health apps in a *mix and match* approach, along with a wide range of other sources including friends, family, and health care professionals (HCPs) [7]. They seek information with a fine balance between awareness and information seeking, with the risk of increasing anxiety and confusion [7]. By accessing apps and digital information during pregnancy, women may be seeking to understand *normality*, to help them to check if their experiences or symptoms are to be expected or something they should take action on [8]. Pregnant women often seek the experiences of others to support their decision-making or to make them feel more informed when raising discussions with professionals [8].

The national child health and well-being charity, Best Beginnings, launched the Baby Buddy app in November 2014 [8]. The charity describes the Baby Buddy app as “an electronically delivered health intervention” that is intended to support and guide women through pregnancy (antenatal period) and the first 6 months of their child’s life (postnatal period). The app was co-designed with mothers to inform and empower women of all ages and from wide sociodemographic backgrounds, so that all expectant parents can search for and benefit from high-quality information and support during pregnancy. Best Beginnings worked closely with HCPs to ensure that the Baby Buddy app provided evidence-based information [8].

Best Beginnings has taken a universal proportionalism approach in designing the app, and, as a child health charity, is committed to embracing the growing digital health space to support young families. The content of the Baby Buddy app is written so that it can be understood by anyone with a reading age of ≥11 years with a read aloud element available and can be downloaded free of charge from the Apple App Store and Google Play Store by anyone with a suitable smartphone. The app uses a blend of the 5 common types of content identified by Peyton and Wisniewski [6], but, in addition, offers a large amount of professional and peer-voiced video content across medical, social, and emotional pregnancy and parenthood experiences. The app also allows for personalization at set up including the names of partners or supporters involved in the parent journey. Although the Baby Buddy app was developed to target young mothers, it was also accessible to a wide range of mothers and families. This was evident from the demographic characteristics captured in the Baby Buddy app downloads [9]. This study is one of the first to evaluate a United Kingdom–based pregnancy app that provides dynamic information and behavior change content, which has been widely embedded in the National Health Service (NHS) maternity services, and to further understand the perceived benefits and challenges of its use.

### Study Objective

An independent, longitudinal, mixed method study of the Baby Buddy app was commissioned by Best Beginnings, funded by the Big Lottery, United Kingdom [10].

This observational study assessed the effectiveness of the Baby Buddy app in improving maternal self-efficacy and mental well-being [10,11]. It also involved a qualitative arm that aimed to understand when, why, and how first-time mothers use the Baby Buddy app and the perceived benefits and challenges [10]. This paper reports on the qualitative arm of the study.

## Methods

### Overview

Appreciative Inquiry (AI) was chosen to underpin the mixed methods to add depth to the quantitative findings [12]. AI is an emerging research methodology that has theoretical and philosophical underpinnings in action research and organizational change [13] and has been used effectively within a variety of complex structures including health and social care settings. It was useful because it focused on the positive, that is, *what works well*, before moving on to *what could be improved.* It involves the systematic discovery of what is most effective [12,14].

### Sample and Recruitment

Recruitment for the qualitative arm was from three maternity units participating in the main BaBBLeS study [10,11] (located in the Northwest, Southeast, and Midlands, England). The inclusion and exclusion criteria are listed in Textbox 1. Each woman received a participant information booklet that combined the study invitation letter and information booklet.

Women who participated in the longitudinal study [10] were also invited to a telephone interview or focus group, and additional informed consent was obtained. Overall, 28% (17/60) of the first-time mothers participated in the qualitative study via telephone interviews (9/17, 53%) or a focus group (8/17, 47%) across the 3 sites, with women ranging from 12 to 37 weeks (3-9 months) of the postnatal period.

**Inclusion and exclusion criteria for participants of the main BaBBLeS study, from which this sample was drawn [15,16].**

**Inclusion criteria**

- Aged ≥16 years
- No previous live child
- Between 12 weeks and 16 weeks and 6 days of gestation

**Exclusion criteria**

- Aged <16 years
- Already has ≥1 child
- Before 11 weeks and 6 days or after 17 weeks of gestation

### Data Collection Tools

One-to-one interviews or focus group interviews were offered and conducted in the postnatal period. A total of 60 women agreed to be contacted for an interview across the recruitment sites. Of these 60 women, 2 (3%) women declined, 9 (15%) were interviewed over the telephone, and 8 (13%) attended the focus group. A breakdown of this sample is presented in Table 1. The focus group was *baby friendly* to accommodate additional needs including a sensory playroom. A flexible interview style with prompts was developed by the research team, and telephone interviews were offered, as these are less demanding in terms of the participants’ time [17]. Interviews were conducted by experienced health researchers, including registered midwives.

Telephone interviews were flexible, which was particularly important when considering that participants were first-time mothers and were interviewed by midwives who were best placed to discuss and question any concerns the participant was having. The interviews were audio-recorded, anonymized, and transcribed verbatim. To maintain consistency and collect comparable data, the interview schedule for the telephone interviews was developed by the research team alongside the schedule used for the focus groups. Drawing on the AI approach, the interviews specifically explored when, why, and how first-time mothers use the Baby Buddy app and the perceived benefits and challenges.

Using the same principles, focus groups were conducted by experienced health researchers, including registered midwives, over a 2-hour period during which the first-time mothers were encouraged to work in groups and discuss among themselves. Consistent with the AI approach [12], comment cards, sticky notes, and colored pens were used to discuss *what worked well* and *what could have been better*. In practice, this meant writing or drawing bullet points to help them to explain their thoughts. Visual prompts included laminated screenshots of the Baby Buddy app’s key features and sections. The Baby Buddy app was also made available on electronic devices, in case they wished to remind themselves of any areas within the app.

**Table 1:** Breakdown of the qualitative sample by site and level of participation (N=60).

Sites	App users who had agreed to be contacted, n (%)	Refusals or noncontactable users, n (%)^a^	Users who were interviewed, n (%)^b^	Users who attended the focus group, n (%)^c^	Total number of users who were interviewed or attended the focus group, n (%)^d^
Northwest	10 (17)	8 (13)	2 (3)	0 (0)	2 (3)
Midlands	38 (63)	26 (43)	4 (7)	8 (13)	12 (20)
Southeast	12 (20)	9 (15)	3 (5)	0 (0)	3 (5)

^a^Total=43/60, 72%.

^b^Total=9/60, 15%.

^c^Total=8/60, 13%.

^d^Total=17/60, 28%.

### Data Analysis

An inductive thematic analysis was conducted based on the in-depth data collated. The thematic analysis was guided by a commonly practiced process described by Braun and Clarke [18]. Principally, this method involves manually sifting through the transcription data, and the codes obtained from that data were related to the study objectives and the AI approach. Transcriptions were read and then reread to ensure in-depth understanding of the meaning behind the written text, and then, relevant phrases were grouped, framed into grids and tables, categorized into themes, and regrouped into *benefits* or *challenges*. Transcripts were reviewed independently by 2 members of the team, with grouping discussed and final themes agreed upon. Once all the relevant text was coded into themes and substantiated with primary evidence, they were recorded in a finalized matrix (refer to sample matrix, *benefits*).

### Ethics Approval and Governance

Ethics approval was obtained from the West Midlands - South Birmingham research ethics committee (reference number 16/WM/002) via the web-based Integrated Research Application System. Ethics approval was also sought from and approved by the research ethics committee at the Faculty of Health and Life Sciences, Coventry University (reference P45795), where the *medium to high risk* procedure was adhered to. All participants were anonymized with unique participant information numbers, and transcription was undertaken to ensure total anonymity.

## Results

### Overview

Consistent with the AI approach, the findings are presented under 2 subheadings, which reflect the structure of the focus group and interviews, where the questions were posed according to these main questions. In terms of discovering when, why, and how first-time mothers interacted with the app, *what worked well* was described to be linked to themes around accessibility of information, including daily pop-ups and a need to seek knowledge on certain aspects of pregnancy and childbirth. Themes around *what could be improved* were about individual preferences regarding app function and content. The perceived benefits were described in terms of access to information considered reliability, and increased sense of confidence to engage in further discussion with HCP and partners. First-time mothers also expressed a desire for great face-to-face engagement with HCPs or peers as a need that the app will not be able to address.

### Benefits

#### What Worked Well?

In describing what worked well, mothers discussed how they used the app to increase their knowledge and understanding of pregnancy topics and app, and some had been guided to do so by HCPs. Although some mothers would use this app along with a range of other sources to seek information about specific topics, the app would prompt them to discover topics that they had not yet considered. There were 4 themes that emerged from the mothers’ accounts of what they thought worked well in using the app.

#### New Information

Throughout the interviews and focus groups, first-time mothers spoke about how they liked acquiring the information they received from the Baby Buddy app and how this made them feel that they had increased their knowledge. They suggested that it was just the right amount of information, topics were concise and easy to understand, and they were able to recall the details they had learned in the focus group and interviews:

I like the information about what size it was and what to think about in terms of my health, and how the baby was growing and getting ready.

P6

The first-time mothers also discussed the fact that the information on the Baby Buddy app gave them more information about their pregnancy, particularly when engaging with HCPs. This was evident, specifically when accessing key words that they may have been unfamiliar with:

So sometimes my midwife would say certain things, and I would like, if it was something medical, go and look at what was going on, and what words meant, and also like for areas, like GPs and different...I’d go and look for information there...

P33

The information provided by the Baby Buddy app continued from pregnancy to postnatal period and may have increased their knowledge. First-time mothers also suggested that it was nice to still have notifications regarding their baby’s development up to a month after childbirth. Breastfeeding was a particular feature from which first-time mothers felt that they had learned a lot:

Gosh there’s loads, I think I learned that you can restart your milk supply, and different ways of feeding her so, I struggled a little bit at first because she was taking too much too quickly, but I read that if I leant back a little bit it would slow the flow down.

P102

From the community, the community one helped quite a lot, when the baby was first born I was told to look at it for like breast feeding bits and stuff, by my midwife and the breast feeding team...that was useful.

P103

Quotes demonstrate how first-time mothers were not only actively using the information they had found to support their decisions in the postnatal period but also how HCPs and first-time mothers were interacting via the Baby Buddy app, which may have helped to keep the lines of communication open. First-time mothers also learned about the size and development of their child throughout pregnancy via the Baby Buddy app, which seemed to support engagement with the app and may improve first-time mothers’ confidence and feeling of being in control:

How much is your baby, for example at 36, you see a picture and your baby, what is doing your baby, with the eyes, the mouth. For vomiting, it tells you when your vomiting will stop, or what you feel when your baby is coming in the last few months.

P104

Probably more about the fetus’s growth, yes what it was actually doing at certain points, as opposed to the birth, because I think a lot of my friends already had children and I’ve done a lot of work with mothers and babies in my job, I think I was quite aware of certain stories and options, yes but it was definitely the fetus’s growth...Which is really exciting to know especially in a first pregnancy isn’t it.

P105

Overall, the data suggest that participants were using the Baby Buddy app both during the antenatal and postnatal periods to access knowledge and inform decision-making during their journey as a first-time mother.

#### Reliability

The themes developed from the extracted quotes indicate that first-time mothers felt one of the clear benefits was that the information in the Baby Buddy app was provided by professional sources, which conveyed reliability, as described in these extracts:

And I think that the Baby Buddy one because you know that it’s been tried and tested by midwives it’s not just generic information, every day you’re getting something to think about.

P58

Yes, because you feel confident knowing that it's tried and tested by midwives, in the videos you have the Midwives perspective, I liked that.

P66

When first-time mothers compared the Baby Buddy app with other web-based sources of information, such as other maternity apps and sources such as Google, they felt that these may not be as reliable. After watching the film footage of midwives providing information on topics such as breastfeeding, first-time mothers described feeling reassured that they were receiving reliable information. Mothers seemed to trust the information they were receiving, which helped to reassure them:

...And at the end of the week it would say the measurements of the child and what kinds of things you should be going through, so it really helped you in the pregnancy just to know what was happening, instead of like panicking about things, so like that’s normal...

P4

These comments suggest that the Baby Buddy app acted as a source of comfort for some first-time mothers, helping them to stay calm and positive about their pregnancy. Participants appreciated the videos with accounts of experiencing motherhood:

Thinking about it I did watch some of the films with the mums talking, about their experiences, I did watch those as well, a few of them, just interesting really, I don’t think I necessarily got anything I didn’t know from it, but it’s nice to see, with other mums.

P14

The Baby Buddy app can be used to set reminders for appointments, dates to remember, and exercise tool that participants reported using:

You could use it to remind yourself to do things, like your pelvic floor exercises.

P29

Yes, and if you have appointment for my baby or my appointment, so I go to the application and I know.

P30

In addition to the app providing information, it also included capabilities that allowed users to add information to the app, such as appointments with midwives. This feature of the app encourages long-term engagement by providing first-time mothers the opportunity to use the app throughout pregnancy and after childbirth. This also suggests that the Baby Buddy app was being used as an organizational tool for first-time mothers, keeping the lines of communication open between them and their HCP.

#### Using the Baby Buddy App—Ease of Access

While exploring when, how, and why first-time mothers used the Baby Buddy app, mothers agreed that, once they had downloaded it, information was easily accessible. They were able to track their daily progress, read daily updates via pop-ups, and find information related to specific topics with ease. They particularly liked the daily pop-ups, which were useful for women who worked and did not have time to search through information regarding their pregnancy, as described in the following quote:

...The updates would come, and they didn’t like override, some apps pregnancy things coming all the time, this one just gave you one simple answer a day, so nice and easy and not too much...

P26

A steady flow of alerts and pop-ups provided by the Baby Buddy app ensured that women had easy accessibility and readily available information throughout their pregnancy, regardless of where they were. When more detail about a particular topic was required, it could be accessed via the app at a time that suited women:

...Because you’re getting information every day, it will provide you with links, the app was the foundational platform for looking up more stuff, it would give you like a summary of what to expect, so one thing I liked about the app was, you didn’t necessarily have to wait to say 7 months pregnant to tell you about pain relief options, it would make suggestions about that early on, so that when I did go to my antenatal classes where they teach you about that stuff I kind of had an idea and then you knew you could ask more questions.

P36

In turn, the alerts and pop-ups led to first-time mothers watching films about specific topics and accessing their customized character or *avatars* (where the appearance, such as hair, skin tone, clothes, and body shape, can be altered to resemble themselves or designed to their preference to guide them through the app content). They also particularly enjoyed videos about breastfeeding, which provided another layer of depth of information:

[Talking about breastfeeding and latching] Yes, people can tell you how to do it, but actually seeing on the video it being done was much clearer.

P65

In particular, some participants also used the Baby Buddy app to find information about pregnancy-related problems. A woman told us that as soon as she was diagnosed with pre-eclampsia, she used the app for information, which helped her as explained in the following quotes:

...The videos I watched were mostly to do with pregnancy, it was actually like really helpful, cos I ended up really ill with the pregnancy, that’s why I’ve got him now, I shouldn’t have had him, he shouldn’t have been here, he was 5 weeks early, I had pre-eclampsia...yes, so that app did help me, it guided me a little bit.

P3

Yes I mean, some of the things that obviously I’m a first time mum, so, no matter how confident you feel going into it, once you’ve given birth its looking for answers to questions that you don’t realize you’re going to have beforehand if you know what I mean?

P2

In addition to being able to access daily information, films, and updates and personalize avatars, the first-time mothers expressed approval for the Baby Buddy app’s sharing capability, which allowed them to share *tailor-made* information with partners and their families and friends:

I like the fact that the app allows you to put your husband’s name in, so it says you can talk to [person's name is removed] about this, and he could have downloaded the app, I thought it was really nice.

P59

...And it refers about your husband or partner by name if you put that information in, I really like that. I would definitely recommend it to other people, my sister’s just got pregnant and I recommended it to her.

P60

These findings indicate that the Baby Buddy app’s prompts, short films, and easily accessible information may be particular features that encouraged women’s engagement with the app. The sharing capability of the app also ensured that women could provide information to their friends and partners, which may have further improved app engagement.

#### Increased Confidence

A further clear benefit was that the more the mothers felt the information they were accessing within the app was reliable, the more they used it to support their pregnancy and postnatal care. First-time mothers felt that being equipped with the knowledge they had accrued from the Baby Buddy app gave them more confidence to plan their care with an HCP:

I think I used it to make choices, I used it together with parent-craft classes, I used them together because I liked that you had the video of the midwife explaining different types of pain relief options, and you had the script that you could read about the different options, so it just meant you could really think about it, one of the things I did was I downloaded the NHS birth-plan template and wrote mine out in pencil so that when went to the actual classes, I could ask questions, I could change my plan, it gave you confidence that you weren’t going off to something with nothing.

P37

Participants from the focus groups also reported that they would use the information from the Baby Buddy app to support their discussions around choice with partners and family members and that they were feeling knowledgeable; therefore, they had the confidence to do so. This may have provided women with more control over their choices and decisions, which, in turn, empowered them to feel more confident about the care they received and the care they provided to their baby:

I just thought it was a good platform, because as a new mum you know you’re pregnant but you don’t know what to expect, so it’s always nice to have this foundational knowledge to make you feel confident about decisions that you are making. It wasn’t complicated to use.

P64

### Challenges—What Could Be Better?

#### An Intervention That Supports Help-Seeking Behavior

Although first-time mothers valued and trusted the information provided by the app, they expressed a preference for face-to-face support from their HCPs. When they needed specific information about breastfeeding or general advice for their child, they would often seek advice from an HCP in preference to accessing the Baby Buddy app’s features:

But if we think we’re doing something wrong then I tend to ring the doctors, or the health visitor...because we’re first-time parents and we don’t want to do anything wrong, and we don’t want to go by the book, like, in case it’s not the right decision, I think it depends on your baby’s development as well I think.

P80

However, data also highlighted that, although first-time mothers enjoyed interacting with the Baby Buddy app, they still preferred reassurance from an HCP. This could be because they preferred face-to-face contact and may have needed further reassurance about the decisions they were making.

First-time mothers also spoke about their preference for antenatal classes toward the latter half of their pregnancy. A participant described that the amount of Baby Buddy app use diminished once antenatal classes were attended:

...Towards the end when I started NCT classes I used it less, but it was definitely very helpful in the first 6 or 7 months I’d say.

P6

Antenatal classes offer the opportunity for facilitated learning and social interaction with peers, which are not available to women through the Baby Buddy app.

#### Where the Need for App Improvements Was Identified

Although first-time mothers spoke positively about the daily information, knowledge, and support they received via the Baby Buddy app, there were particular areas throughout pregnancy and after childbirth, in which participants would have liked more detail, including pregnancy and labor progress, birth plans, checklist of items to have ready for giving birth, and long support after birth:

The thing I was sad about with the Baby Buddy app was that it finished...it would be nice if it followed you for one year after you’ve had your baby, because it’s a really useful simple app.

P66

First-time mothers suggested that expanding the detailed information for the week-by-week pregnancy journey will be beneficial, specifically further information about their child’s development and what to expect:

The only things that I would have liked more of was like the week by week update, as they’re growing, the fetus, a bit more detail in it.

P92

First-time mothers also suggested additional features for the Baby Buddy app such as tools to make decisions about labor, contraction counter, and accessible birthing plan template. Nevertheless, in more than two-thirds of interviews, the mothers did not think that anything needed improvement in the Baby Buddy app. Some first-time mothers reported downloading the Baby Buddy app, but did not actively use its features. This may be owing to individual differences between first-time mothers who participated in this study. This includes language considerations, because some mothers struggled to fully interpret or understand the information, as there was no option apart from English language:

If you could put in this application the Greek language, or different languages, you know, because sometimes I can’t read it, I can’t understand it, sometimes I need to copy paste and translate. So put in more languages. I have friends from Poland, and they use this application and they tell me to translate for them, and I may be working or something like that.

P96

Mothers in this study may also not have used the Baby Buddy app after downloading it because of their existing knowledge and beliefs. This was particularly evident when they were asked whether the information and films on breastfeeding had helped to guide their decisions around the topic area:

I always knew that breastfeeding was what I wanted to do, but it set my mind in that mindset, this is what I’m going to do, I was determined to do it.

P97

## Discussion

### Principal Findings

This is one of the first qualitative studies aiming to understand when, why, and how first-time mothers use the Baby Buddy app and the perceived benefits and challenges. By taking an AI approach, the questions about what worked well and what could be improved were explored with app users who were able to relate a range of experiences they had with using the app throughout their pregnancy to postnatal journey.

First-time mothers who participated in the qualitative arm of the study found that the Baby Buddy app worked well owing to its accessibility and that the information was concise and easy to find. This made them feel that they had gained knowledge, and they felt that the information was reliable owing to its association with HCPs in the film and written content. They liked that although the information was linear, different aspects could be accessed as and when needed. They particularly liked the personalized daily updates about the progress of their pregnancy and child development. This had a snowball effect in that it reassured these mothers that the information they were receiving was correct. This appeared to increase their confidence in using the app and encouraged them to continue using it throughout pregnancy and postbirth care with the HCP. These mothers were particularly reassured by the reliability of the sources of the videos in Baby Buddy that featured midwives. This gave them the confidence to ask more detailed questions to their HCPs, knowing that they could always refer to the Baby Buddy app if necessary. Time pressure is a known key barrier to effective discussion about the content of leaflets [19]; the Baby Buddy app offers women the opportunity to browse and explore information according to their own needs and interest. This mitigates the selectivity of HCPs and may contribute to a sense of increased confidence in discussing birth options.

Our findings align with other studies that have explored how women use digital information to enhance their candidacy for appropriate use and escalation of concerns to an HCP [20]. Similarly, we found that women were more confident in discussing issues with HCPs when they felt that they had some knowledge as a starting point. Candidacy in the context of maternity care has been described as a dynamic and continuous process subject to redefinition through interactions [21]. Eligibility for health care is determined by the individual and health services, and there may be increased barriers for a more vulnerable population [21]. The concept of empowerment is one that is frequently referred to within maternity services, and the external attributes of this concept include access and control over resources, such as reliable knowledge and information [22]. The nurturing of candidacy and empowerment within the maternity population is a critical part of the transition to parenthood; the consequences can influence satisfaction with birth experiences, contribution to overall health of families, and development of self-advocacy [22]. An interesting novel observation emerging from our findings was that women reported using the information from the app to support conversations with family members about birth and feeding choices. The influence of people in their immediate social sphere are known to have significant influence on mothers’ decision-making [23]. The participants were recruited from the BaBBLeS cohort study [10,11], which had change in self-efficacy as the primary outcome. Although the study reported no significant difference in change in self-efficacy scores from pregnancy to 3 months after birth in app users, it is worth noting that average baseline self-efficacy (measured using the Tool to Measure Parenting Self-Efficacy) was high. Mobile health (mHealth) adoption and engagement in users with high baseline self-efficacy have been reported in other studies in mHealth [15]. In our findings, women described how access to reliable digital information had provided “a foundational knowledge to make you feel confident about decisions you are making.” Self-efficacy theory [16] implies that the antecedents of self-efficacy include mastery and vicarious experience, both of which can be observed from the participants’ experiences of using the app. This aligns with previous studies in which women who engaged in web-based forums demonstrated increased health literacy and further confidence in raising questions with health care providers [13,24].

It should be noted that the first-time mothers who were interviewed were often using multiple sources to access information, including other maternity apps or web pages. They used a *pick and choose* technology consumer style approach to decide the particular type of information that they used from different apps. This is in agreement with an investigation into mHealth activities of pregnant women who are disadvantaged in the United States, which discovered that a high number of women (97%) engaged in pregnancy health information through a combination of websites and apps [25]. This is further evidence that women turn to a wide range of both web-based and offline resources for informational needs during pregnancy [7]. This again highlights the need for reliable and consistent information in mHealth interventions during pregnancy, as confusion and contradicting or false information can undermine the relationship with the HCP, lead to erosion of candidacy, and have negative impacts. Although first-time mothers appreciated the information they received, many preferred in-practice supports via HCPs, such as their midwife. Although they felt that the Baby Buddy app was reliable, as first-time mothers, they wanted to seek face-to-face advice from an HCP to support their decision-making. This highlights the central importance of *a face* within the health system [26].

All data for this study were collected before the 2020 COVID-19 pandemic, when, to limit contact with pregnant women, antenatal care provision was conducted remotely. Feedback to evaluate a virtual antenatal clinic implemented at this time in the United Kingdom reported that 86% of service users were highly satisfied with the virtual clinic. Notably, there were no differences by demographics of either age or ethnicity, but 56% of the people still preferred face-to-face clinics [27]. HCPs also reported high level of approval for long-term use of virtual clinics, with 78% rating their experience as the same or better than face-to-face clinics [27]. Although the COVID-19 pandemic has pushed digital and remote solutions to the fore and they were received well as a suitable crisis contingency, time will tell how much of reduction in face-to face contact is acceptable to women outside the context of a global pandemic. Although virtual clinics offer feasible advantages to women in terms of reducing travel, time away from work, and childcare challenges, this must be considered in the context of those for whom digital exclusion is a reality, who experience language and context difficulties in virtual encounters, and who may need face-to-face appointments to build a relationship of trust with their care team. As England rolls out a Midwifery Continuity of Carer model, with personalized and relationship-based care at its core [28], the place of digital and virtual care is already being considered alongside maternity digital transformation. A key aspect of the application of mHealth to support midwifery tasks has the potential to lead to efficiencies that free up time for relationship-building in face-to-face contact.

One of the barriers was that some mothers made a conscious decision not to engage in some of the more “nerve wracking” topics of information, such as labor or care after birth. The extent to which users of web-based content can exert this avoidance selectivity should be considered by HCPs when discussing any web-based resource use. This may also help to increase discussion around any fears, worries, or concerns they may have; highlight mental health issues; and seek further support if needed. It has previously been shown that people who do not engage in pregnancy health apps had scored higher on the State Trait Anxiety Index than those who engaged [5]. In the main cohort study from which the sample for this study was drawn, low levels of self-reported social support were associated with poor mental well-being scores [29]. In addition to this, a negative association was seen between self-reported social support and socioeconomic status [29]. An investigation into how women narrate anxiety and depression during pregnancy found that women are highly conscious of the socially constructed imperative to be happy and positive to maintain the perception of a *good mother* [30]. Women distanced themselves from the language of distress and wished to appear in control of their emotions [30]. This included using discursive strategies such as distancing from the depressed self, using codes to discuss issues around distress, and seeking to portray balance and control of emotions to maintain the *happiness imperative* [30]. Future app design and studies on the human-computer interaction in a pregnancy context should explore this issue in more depth to understand how information can be presented to avoid increasing anxiety, feelings of social exclusion, or perpetuation of societal ideal imagery.

At the time this study was conducted, the Baby Buddy app lacked social engagement within its features. First-time mothers felt that during the latter half of pregnancy, antenatal classes were more engaging and thus provided the opportunity to speak to other first-time mothers about their decisions and worries in addition to HCPs. Having access to peer support via social groups, shared discussion and perspectives may increase the likelihood of mothers feeling “safer” to interact about these topics. A recommendation for the future use of any app will include conspicuous information about where to find group support sessions with other first-time mothers. Although the Baby Buddy app potentially has this feature, this study found that many mothers were not aware, and thus, it was undiscovered. In addition, an interactive web-based group discussion feature to keep first-time mothers engaged throughout later stages of pregnancy and after birth may be particularly valuable to women during COVID-19 recovery but will need additional resources for content moderation. Further studies on women’s preference for group antenatal education via digital platforms, face-to-face mode, or a combination of both is also required for future service design, including full evaluation of innovations emerging from the pandemic [31]. It is worth noting that since this study was conducted, the Baby Buddy app has been developed further with additional content including increased information on local services, information for fathers, and access to a maternity helpline.

Although this study interviewed only 17 women, the team gathered many rich and in-depth views and perspectives from first-time mothers. The extent to which these findings will apply to second-time or third-time mothers is difficult to infer. Although other studies have also found first-time mothers to be more frequent app users [5], they may have different needs and expectations, given previous experience with maternity services. The cohort study from which our sample was drawn [10] was typical of a UK pregnancy and digital health research demographic in that it was predominantly White, well-educated, and older women who volunteered for the study, despite the recruitment sites representing a broad demographic. Strategies to ensure engagement of broad ethnic, socioeconomic, and maternal age groups will provide great understanding of the transferability of the findings. Studies focusing on specific demographic groups and their interaction with digital services and interventions is required to produce a truly equitable future maternity service, which undoubtedly will comprise a combination of digital and face-to-face delivery of care. Similar studies exploring the experiences of fathers and other parenting dyads in their perinatal mHealth needs will be of value, including the need for inclusion of lesbian, gay, bisexual, transgender, queer, and other communities in the perinatal digital space.

### Conclusions

This paper adds to current literature on pregnancy app interaction and explores how women have engaged with a specific NHS-approved app that was co-designed with service users and has sought professional validation of content for reliability. The obtained insight builds on the understanding of how mHealth and digital interventions can contribute to women’s sense of empowerment through access to information that can be used to negotiate health care choices, both with health professionals and within families. Before the COVID-19 pandemic, first-time mothers preferred in-practice support from their HCPs and engaged less with the app as they developed peer support in later pregnancy from antenatal groups. Further studies are required to determine how these preferences evolve after the pandemic. Although the Baby Buddy app was useful, multiple sources of web-based information was sought by first-time mothers when it was not available in the app.

Findings presented in this paper can support the design and development of interventions aimed to encourage first-time mothers’ engagement with digital health management tools. In addition, these findings can lead to further understanding of mHealth behavior during pregnancy and the relationship between digital technology and human interaction.

## Abbreviations

AI: Appreciative Inquiry
HCP: health care professional
mHealth: mobile health
NHS: National Health Service
